# Identification of Platform-Independent Diagnostic Biomarker Panel for Hepatocellular Carcinoma Using Large-Scale Transcriptomics Data

**DOI:** 10.3389/fgene.2019.01306

**Published:** 2020-01-10

**Authors:** Harpreet Kaur, Anjali Dhall, Rajesh Kumar, Gajendra P. S. Raghava

**Affiliations:** ^1^ Bioinformatics Center, CSIR-Institute of Microbial Technology, Chandigarh, India; ^2^ Department of Computational Biology, Indraprastha Institute of Information Technology, New Delhi, India

**Keywords:** liver cancer, hepatocellular carcinoma, biomarker, expression, diagnosis, survival, machine learning, classification

## Abstract

The high mortality rate of hepatocellular carcinoma (HCC) is primarily due to its late diagnosis. In the past, numerous attempts have been made to design genetic biomarkers for the identification of HCC; unfortunately, most of the studies are based on small datasets obtained from a specific platform or lack reasonable validation performance on the external datasets. In order to identify a universal expression-based diagnostic biomarker panel for HCC that can be applicable across multiple platforms, we have employed large-scale transcriptomic profiling datasets containing a total of 2,316 HCC and 1,665 non-tumorous tissue samples. These samples were obtained from 30 studies generated by mainly four types of profiling techniques (Affymetrix, Illumina, Agilent, and High-throughput sequencing), which are implemented in a wide range of platforms. Firstly, we scrutinized overlapping 26 genes that are differentially expressed in numerous datasets. Subsequently, we identified a panel of three genes (*FCN3, CLEC1B*, and *PRC1)* as HCC biomarker using different feature selection techniques. Three-genes-based HCC biomarker identified HCC samples in training/validation datasets with an accuracy between 93 and 98%, Area Under Receiver Operating Characteristic curve (AUROC) in a range of 0.97 to 1.0. A reasonable performance, i.e., AUROC 0.91–0.96 achieved on validation dataset containing peripheral blood mononuclear cells, concurred their non-invasive utility. Furthermore, the prognostic potential of these genes was evaluated on TCGA-LIHC and GSE14520 cohorts using univariate survival analysis. This analysis revealed that these genes are prognostic indicators for various types of the survivals of HCC patients (e.g., Overall Survival, Progression-Free Survival, Disease-Free Survival). These genes significantly stratified high-risk and low-risk HCC patients (p-value <0.05). In conclusion, we identified a universal platform-independent three-genes-based biomarker that can predict HCC patients with high precision and also possess significant prognostic potential. Eventually, we developed a web server HCCpred based on the above study to facilitate scientific community (http://webs.iiitd.edu.in/raghava/hccpred/).

## Introduction

Cancer is a heterogeneous disease driven by genomic and epigenomic changes within the cell (Sharma et al., 2010; Dawson and Kouzarides, 2012; Nagpal et al., 2015; Flavahan et al., 2017; Kamel and Al-Amodi, 2017; Chatterjee et al., 2018; Kagohara et al., 2018; Narrandes and Xu, 2018; Nebbioso et al., 2018; Kumar et al., 2019). Gene dysregulation is considered a hallmark of cancer. Among the 22 common cancer type, hepatocellular carcinoma (HCC) ranks at sixth in terms of frequency of occurrence and fourth at cancer-related mortality (Siegel et al., 2019). The etiology of HCC can be induced by multiple factors, especially hepatitis viral infection, alcoholic cirrhosis, and consumption of aflatoxin-contaminated foods (Ho et al., 2016). Although various traditional and locoregional treatment strategies such as hepatic resection (RES), percutaneous ethanol injection (PEI), radiofrequency ablation (RFA), microwave ablation (MWA), and trans-arterial chemotherapy infusion (TACI) have improved the survival rate, patients with HCC still have a late diagnosis and poor prognosis (Tian et al., 2018).

In the past, several studies focus on the identification of biomarkers by comparing the global gene expression changes between cancer tissue and non-tumorous tissues (Shirota et al., 2001; Jia et al., 2007; Marshall et al., 2013; Gao et al., 2015; Kang et al., 2015; Liu et al., 2015; Emma et al., 2016; Komatsu et al., 2016; Cai et al., 2017; Li et al., 2017; Zhang et al., 2017; Li et al., 2018b; Liao et al., 2018; Meng et al., 2018; Wang et al., 2018; Xu et al., 2018; Zheng et al., 2018; Cai et al., 2019; Jiao et al., 2019; Xia et al., 2019; Zhang et al., 2019). Such analyses yield hundreds or thousands of gene signature that are differentially expressed in cancer tissue compared to normal tissue, thus making it difficult to identify a universal subset of genes that play a crucial role in neoplastic transformation and progression (Rhodes et al., 2004). The lack of concordance of signature genes among different studies and extensive molecular variation between the patient’s samples restrains the establishment of the robust biomarkers, promising targets and their experimental validation in clinical trials (Vasudevan et al., 2018). The transcriptome signatures have yet to be translated into a clinically useful biomarker, which may be due to a lack of their satisfactory validation performance on independent patient’s cohort.

In this regard, treatment of HCC remains unsatisfying as only diagnostic and prognostic biomarkers alpha-fetoprotein (AFP) has been established so far. Several other biomarkers AFP-L3, osteopontin, and glypican-3 are currently being under investigation for the early diagnosis of HCC patients (Ocker, 2018). Advancement in the genomics has created rich public repositories of microarray and high throughput datasets from numerous studies such as The Cancer Genome Atlas (TCGA) (Cancer Genome Atlas Research Network et al., 2013), Genomic Data Common (GDC), and Gene Expression Omnibus (Grossman et al., 2016), (Barrett et al., 2013), which provide the opportunity to study the various aspects of cancer. Thus, novel methods exploring the computational approach by merging multiple datasets from different platforms could provide a new way to establish a robust and universal biomarker for disease diagnosis and prognosis with increased precision and reproducibility. Recently, this approach has been used for biomarker identification of pancreatic adenocarcinoma (PDAC) (Bhasin et al., 2016; Klett et al., 2018). However, various studies employed large-scale data or meta-analysis approaches to identify protein and miRNA expression-based biomarker for HCC diagnosis (Ji et al., 2016; Ding et al., 2017; Chen et al., 2018b; Ji et al., 2018). But, to the best of our knowledge, RNA-expression data are not explored in this regard for identification of the robust biomarker for HCC diagnosis and prognosis.

In order to overcome the limitations of existing methods, we made a systematic attempt to identify genetic biomarkers for HCC diagnosis that apply to a wide range of platforms and profiling techniques. One of the objectives of this study is to identify robust gene expression signatures for discrimination of HCC samples by the integration of multiple transcriptomic datasets from various platforms. Here, we have collected and analyzed a total of 3,981 samples from published datasets, out of which 2,316 and 1,665 are of HCC and normal or non-tumorous tissue samples, respectively. From this, we identified 26 genes, which are commonly differentially expressed in uniform patterns among most of the datasets, which provides a universally activated transcriptional signatures of HCC cancer type. Further, we have established a robust “three-genes-based HCC biomarker” implementing different machine learning techniques to distinguish HCC and non-tumorous samples with high precision. Additionally, the survival analysis of HCC patient’s cohorts using these genes revealed their significant prognostic potential in the stratification of high-risk and low-risk patient’s groups. To the best of our knowledge, this is the first study regarding HCC cancer type for the identification of universal platform-independent diagnostic biomarkers by integrating data from multiple platforms implementing machine learning approaches.

## Materials and Methods

### Dataset Collection

#### Collection of Gene Expression Datasets of HCC

In this study, we extract raw expression data of 30 datasets, where 29 transcriptome datasets were obtained from GEO and one is from TCGA; each dataset contains at least 10 samples. The following is the list of datasets obtained from GEO: GSE102079 (Chiyonobu et al., 2018), GSE22405, GSE98383 (Diaz et al., 2018), GSE84402 (Wang et al., 2017), GSE64041 (Makowska et al., 2016), GSE69715 (Sekhar et al., 2018), GSE51401, GSE62232 (Schulze et al., 2015), GSE45267 (Chen et al., 2018a), GSE32879 (Oishi et al., 2012), GSE19665 (Deng et al., 2010), GSE107170 (Diaz et al., 2018), GSE76427 (Grinchuk et al., 2018), GSE39791 (Kim et al., 2014), GSE57957 (Mah et al., 2014), GSE87630 (Woo et al., 2017), GSE46408, GSE57555 (Murakami et al., 2015), GSE54236 (Villa et al., 2016; Zubiete-Franco et al., 2019), GSE65484 (Dong et al., 2015), GSE31370 (Seok et al., 2012), GSE84598, GSE89377, GSE29721 (Stefanska et al., 2011), GSE14323 (Mas et al., 2009), GSE25097 (Lamb et al., 2011; Tung et al., 2011; Wong et al., 2016), GSE14520 (Roessler et al., 2010; Zhao et al., 2015), GSE36376 (Lim et al., 2013), GSE36076). All GEO datasets were obtained using GEOquery package of Bioconductor in R-3.5.3. The TCGA RNA-seq dataset of TCGA-LIHC was downloaded using gdc-client from the GDC data portal. All datasets were curated manually to remove all non-human samples and ensured that only human tissue samples remain in the dataset. Besides, Probe ID mapped to gene symbols extracted from respective platform file and incorporated in the dataset matrix for each dataset. It has been observed that two datasets, i.e., GSE102079 and GSE64041, have three types of samples (HCC, adjacent non-tumor, and normal healthy). Thus, we derived two datasets from GSE102079, called GSE102079_D1 (contains HCC and adjacent non-tumor samples) and GSE102079_D2 (contains HCC and healthy normal samples). Similarly, we derived GSE64041_D1 and GSE64041_D2 datasets from GSE64041. Finally, we derived 32 datasets from original 30 datasets as we derived four datasets corresponding to GSE102079 and GSE64041. Notably, we used one non-invasive dataset (GSE36076), which contains 20 blood samples of peripheral blood mononuclear cells (PBMCs) to evaluate our models.

#### Pre-Processing of Datasets

Each retrieved raw dataset (**Supplementary Data**) was subjected to a detailed curation process. We have pre-processed dataset matrix individually from each profiling technique for different platforms in a standardized manner. In case of Affymetrix datasets, raw data files were pre-processed with background correction; RMA values were calculated using the Oligo package (Carvalho and Irizarry, 2010). In case of Illumina datasets, raw files were processed using Limma and Lumi packages (Du et al., 2008; Ritchie et al., 2015) and finally log2 values calculated using in-house R scripts. Similarly, raw Agilent-1-color and Agilent-2-color files were pre-processed using Limma package individually, then A-values were generated, which were further transformed to log2 values. Eventually, the average of multiple probes computed that correspond to a single gene for each dataset individually employing in-house R scripts. TCGA-LIHC dataset contains FPKM values, which were further converted to log2 values. Entrez transcript IDs were mapped to the gene symbols using GENCODE v22.

#### Datasets for the Identification of Differentially Expressed Genes

**Figure1:**
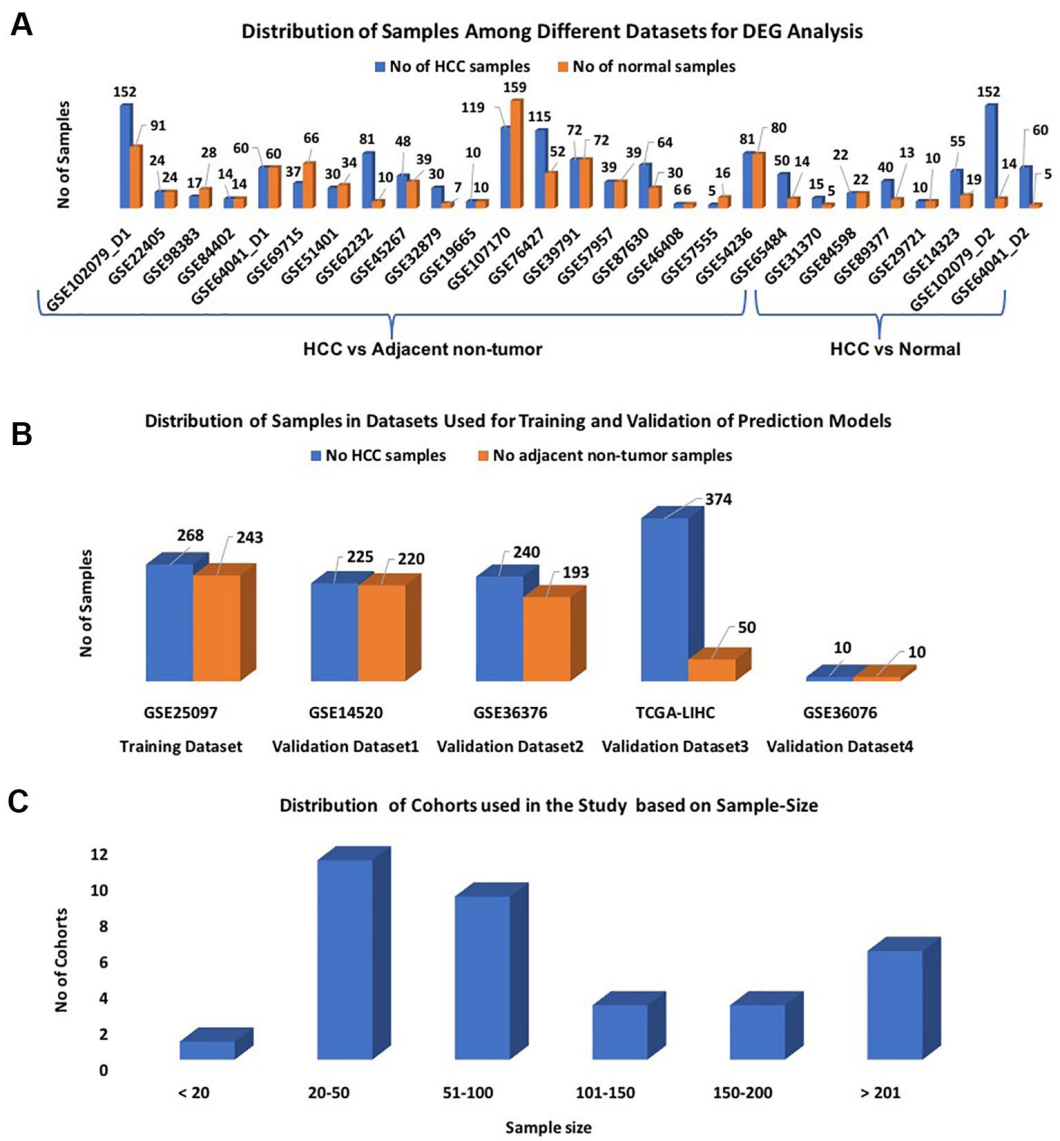
Distribution of samples among datasets used in the study: **(A)** Datasets used for DEG analysis; **(B)** Datasets used for Development of Prediction models; **(C)** Sample-wise distribution of the datasets.

We divide our datasets into two parts: i) datasets for features extraction and ii) datasets for the development of the prediction models. Twenty-seven out of 32 datasets were selected for identification of differentially expressed genes (DEGs); each dataset contains more than 10 samples (**Figure 1A**). These 27 datasets were derived from 25 original GEO datasets. Out of them, 20 datasets contain HCC v/s adjacent non-tumor samples and 7 datasets contain HCC v/s healthy samples. These datasets encompass a total of 1,199 HCC and 949 normal or adjacent non-tumor samples.

#### Training and Validation Datasets

In this study, the GSE25097 dataset was used as a training dataset to develop prediction models; it contains 268 HCC and 243 non-tumor samples (**Figure 1B**). The performance of these models was evaluated on the following three datasets: GSE14520, GSE36376, and TCGA-LIHC, and called them as external validation datasets. As shown in **Figure 1B**, each dataset has a minimum of 400 samples. The distribution of all cohorts used in the current study based on sample size is shown in **Figure 1C**. To validate the performance of models on the non-invasive specimen, we also evaluated the performance on the GSE36076 dataset. This dataset contains 20 blood samples of PBMCs; it contains 10 HCC and 10 healthy individuals. In order to reduce the cross-platform artifacts, we performed quantile normalization using the PreprocessCore library of Bioconductor (Grossman, et al., 2016) package, for each dataset as well as for each profiling technique. This approach is well-adapted in the literature (Huang and Qin, 2018; Klett et al., 2018; Pedersen et al., 2018). These datasets contain a total of 1,117 HCC and 716 adjacent non-tumor samples.

### Identification of Differentially Expressed Genes

Each gene in 32 datasets was analyzed for differential expression using Student’s *t-*test (Welch *t*-test and Wilcoxon *t*-test). It is implemented using in-house R scripts after the assignment of samples to the respective class, i.e., cancer or normal. These tests have been applied previously in different studies for the identification of DEGs (WELCH, 1947; Akaiwa et al., 1999; Carvalho and Irizarry, 2010; Aino et al., 2014; Schulze et al., 2015; Best et al., 2016; Bhasin et al., 2016; Bhalla et al., 2017; Cai et al., 2017; Bhalla et al., 2019; Cai et al., 2019; Kaur et al., 2019). Wilcoxon T-test is used for paired samples and Welch T-test is used for unpaired samples. Only those sets of genes chosen to define DEGs that are statistically differentially expressed between two classes of samples with Bonferroni adjusted p-value less than 0.01. In order to identify a set of differential expression signatures or “core DEGs of hepatocellular carcinoma,” DEGs in all 27 datasets were compared. Finally, only those overlapping genes were considered as “core DEGs of hepatocellular carcinoma,” which have significant differential expression in at least 80% of cohorts. A similar type of approach was previously implemented in various studies (Bhasin et al., 2016; Klett et al., 2018; Li et al., 2018a).

### Identification of Robust Biomarkers for HCC Diagnosis

#### Ranking and Selection of Features

To reduce the number of genes from the selected set of signature, i.e., “the core genes of hepatocellular carcinoma,” genes were ranked on training dataset (GSE25097) using a simple threshold-based approach (Bhalla et al., 2017; Bhalla et al., 2019; Kaur et al., 2019). In the threshold-based approach, genes with a score above the threshold are assigned to cancer if it is found to be upregulated in cancer and otherwise normal; whereas sample is assigned to normal if the gene is downregulated in cancerous condition. We compute the performance of each gene based on a given threshold and identify the top 10 features having the highest performance. We further identified the top 5 features, which give the best performance when evaluated on the training dataset using a 10-fold cross-validation technique. Features were further reduced from five to four and then four to three using a wrapper-based approach. In this technique, one-by-one each feature is removed, and the prediction model is developed using the remaining features. Finally, a combination of features that give the best performance is selected. This technique is also known as the feature-reduction technique.

#### Development of Prediction Models

Here, we have developed the prediction models to distinguish HCC and non-tumorous samples using selected features. These models were implemented using Python package Scikit-learn (Pedregosa et al., 2011). A wide range of machine learning techniques have been used for developing these prediction models that include ExtraTrees (ETREES), Naive Bayes, K-nearest neighbor (KNN), Random Forest, Logistic Regression (LR), and SVC-RBF (radial basis function). The optimization of the parameters for the various classifiers was done by using a grid search with AUROC curve as scoring performance measure for selecting the best parameter.

### Performance Evaluation of the Prediction Models

In the current study, both internal and external validation techniques were employed to evaluate the performance of models. First, the training dataset is used to develop prediction models and standard 10-fold cross-validation is used for performing internal validation, which is commonly employed in the literature (Burton et al., 2012; Bastani et al., 2013; Kourou et al., 2015; Bhalla et al., 2017; Jiang et al., 2018; Bhalla et al., 2019; Kaur et al., 2019). It is important to evaluate the realistic performance of the model on the external validation dataset, which should not be used for training and testing during model development. Therefore, we evaluated the performance of our models on four independent gene-expression cohorts that include GSE14520, GSE36376, GSE36076, and TCGA-LIHC obtained from GEO and The Cancer Genome Atlas (TCGA) (see **Figure 1B**), which were not used for training. In order to measure the performance of models, we used both threshold-dependent and threshold-independent parameters. In the case of threshold-dependent parameters, we measure sensitivity, specificity, accuracy, and Matthew’s correlation coefficient (MCC) using the following equations.

(1) $$Sensitivity\;\left(Sen\right)=\frac{TP}{TP+FN}\times 100$$

(2) $$Specificity\;\left(Spec\right)=\frac{TN}{TN+FP}\times 100$$

(3) $$Accuracy\;\left(Acc\right)=\frac{TP+TN}{TP+FP+TN+FN}\times 100$$

(4) $$MCC=\frac{\left(TP\times TN\right)-\left(FP\times FN\right)}{\sqrt{\left(TP+FP\right)\left(TP+FN\right)\left(TN+FP\right)\left(TN+FN\right)}}$$

where FP, FN, TP, and TN are false positive, false negative, true positive, and true negative predictions, respectively.

In case of threshold-independent measures, we used a standard parameter Area under the Receiver Operating Characteristic (AUROC) curve. The AUROC curve is generated by plotting sensitivity or true positive rate against the false positive rate (1-specificity) at various thresholds. Finally, the area under the curve is calculated to compute a single parameter called AUROC.

### Prognostic Potential of Identified HCC Diagnostic Biomarkers

The prognostic potential of the *“three-genes HCC biomarker”* was analyzed using gene-expression data of TCGA-LIHC and GSE14520 cohorts. The TCGA and GSE14520 datasets contain 374 and 219 tumor samples, respectively. Their clinical information was extracted from GEO, GDC, and the literature (Roessler et al., 2010; Liu et al., 2018a). The clinical characteristics of patients are given in **Table S1** (**Supplementary Information File 1**). Univariate survival analyses and risk assessments were performed by survival package in R (Therneau and Grambsch, 2000; Therneau, 2013). The distribution of the survival risk groups is done by using a log-rank test, eventually represented in the form of Kaplan-Meier plots. A p-value < 0.05 was considered the cut-off to describe the statistical significance in all survival analyses. Here, we analyzed four types of survivals, i.e., OS (Overall Survival), DSS (Disease-Specific Survival), DFS (Disease-Free Survival), and PFS (Progression-Free Survival) for TCGA-LIHC cohort, and two types of survivals, i.e., OS and RFS (Recurrence-Free Survival) (also called as DFS) for GSE14520 cohort. Besides, genes from the signature, univariate survival analysis is also performed on clinical characteristics of patients like age, gender, and tumor stage individually. Additionally, multivariate survival analysis was performed to assess the combined effect of clinical characteristics with the signature genes.

### Functional Annotation of Signature Genomic Markers

In order to discern the biological relevance of the signature genes, enrichment analysis is performed using Enrichr (Kuleshov et al., 2016). Enrichr executes Fisher exact test to identify enrichment score. It provides Z-score and adjusted p-value, which is derived by applying correction on a Fisher exact test. We have considered only those Gene Ontology (GO) terms that are significantly enriched with adjusted p-value less than 0.05.

## Results

### Overview

**Figure 2:**
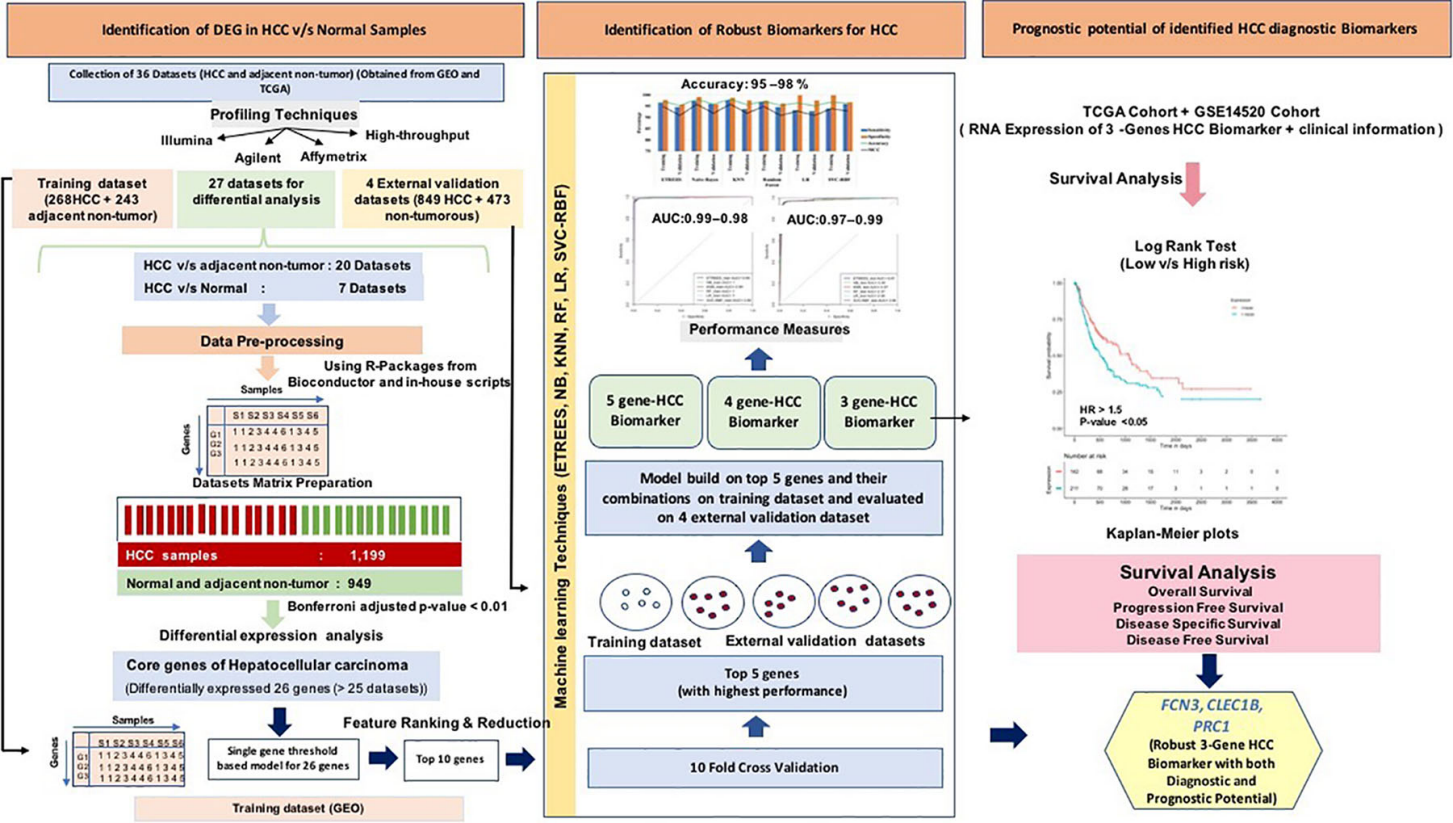
Overview for the analysis implemented in the study.

The pipeline of our analysis is illustrated in **Figure 2**. The detail of each step is described below.

### Transcriptomic Cores for Hepatocellular Carcinoma

#### Identification of the Transcriptomic Cores

The individual statistical differential expression analyses of 27 gene-expression datasets resulted in the identification of hundreds of DEGs (**Supplementary Figure 1**). The 9,954 genes are present among each of the 27 datasets (**Supplementary Information File 1**, **Table S2**). Further, the comparative analysis among all 27 datasets scrutinized 26 overlapping genes that are differentially expressed in 80% or more datasets, i.e., 22 datasets. We called these genes as “core genes for hepatocellular carcinoma.” Among these 26 genes, 12 are downregulated and 14 are upregulated in HCC in comparison to normal samples. The regulatory patterns of the core genes were consistent among most of the datasets (**Table 1**). Additionally, the expression pattern of these genes in training and three external validation datasets is shown in **Figure S2** (**Supplementary Information File 2**).

**Table1:** List of overlapping 26 genes that are differentially expressed (Core DEGs for HCC) between HCC and adjacent normal or adjacent non-tumor samples with Bonferroni p-values < 0.01.

**Gene**	**#Up**	**#Down**	**#Sig**	**#Non-sig**	**Up (%)**	**Down (%)**	**Sig (%)**	**Regulation**
***FCN3***	1	26	24	3	3.70	96.30	88.89	Down
***CLEC4M***	2	25	24	3	7.41	92.59	88.89	Down
***FCN2***	2	25	24	3	7.41	92.59	88.89	Down
***MARCO***	3	24	22	5	11.11	88.89	81.48	Down
***CRHBP***	2	25	22	5	7.41	92.59	81.48	Down
***CFP***	2	25	22	5	7.41	92.59	81.48	Down
***STEAP3***	2	25	25	2	7.41	92.59	92.59	Down
***HGFAC***	4	23	22	5	14.81	85.19	81.48	Down
***CLEC1B***	2	25	23	4	7.41	92.59	85.19	Down
***CXCL12***	3	24	24	3	11.11	88.89	88.89	Down
***MT1E***	3	24	24	3	11.11	88.89	88.89	Down
***NSUN5***	25	2	24	3	92.59	7.41	88.89	Down
***MCM7***	24	3	24	3	88.89	11.11	88.89	Up
***MCM3***	24	3	24	3	88.89	11.11	88.89	Up
***ITGA6***	24	3	24	3	88.89	11.11	88.89	Up
***SSR2***	24	3	23	4	88.89	11.11	85.19	Up
***STMN1***	23	4	24	3	85.19	14.81	88.89	Up
***PRC1***	24	3	23	4	88.89	11.11	85.19	Up
***POLD1***	24	3	23	4	88.89	11.11	85.19	Up
***PBK***	24	3	24	3	88.89	11.11	88.89	Up
***IGSF3***	22	5	23	4	81.48	18.52	85.19	Up
***DTL***	24	3	22	5	88.89	11.11	81.48	Up
***ZWINT***	24	3	22	5	88.89	11.11	81.48	Up
***SPATS2***	24	3	24	3	88.89	11.11	88.89	Up
***GPSM2***	23	4	23	4	85.19	14.81	85.19	Up
***COL15A1***	24	3	22	5	88.89	11.11	81.48	Up

Up, Upregulated in cancer or HCC; Down, Downregulated in cancer or HCC; #Up: No. of datasets in which gene is overexpressed; #Down: No. of datasets in which gene is under-expressed; #Sig: No. of datasets in which gene is significantly differentially expressed; #Non-Sig: No. of datasets in which gene is not significantly differentially expressed; Up (%): Percentage of datasets in which gene is overexpressed; Down (%): Percentage of datasets in which gene is underexpressed; Sig (%): Percentage of datasets in which gene is significantly differentially expressed.

#### Gene Enrichment Analysis of the Transcriptomic Cores

Gene enrichment analysis of these “core genes of HCC” revealed their biological significance. The proteins encoded by the downregulated genes mainly enriched in complement activation and lectin pathways related processes. These genes negatively regulate cellular extravasation. They are also enriched in GO molecular functions like serine-type endopeptidase, oxidoreductase, RNA methyltransferase activity, etc. (**Supplementary Information File 2**, **Figure S3**). Whereas, upregulated core genes are enriched in cell cycle GO biological processes like mitotic spindle organization and mitotic sister chromatid segregation, DNA synthesis and DNA replication, post-replication repair and cellular response to DNA damage stimulus, etc. They are also enriched in GO molecular functions such as exodeoxyribonuclease activity, GDP-dissociation inhibitor activity, DNA polymerase activity and insulin-like growth factor binding, etc. (**Supplementary Information File 2**, **Figure S3**).

### Identification of HCC Biomarkers and Development of Prediction Models

#### Single-Gene Based Prediction Models

All 26 DEGs were ranked on the training dataset using threshold-based approach; ranking is based on their discriminatory power to distinguish HCC from non-tumorous samples (Bhalla et al., 2017; Kaur et al., 2019). The performance of the top 10 genes having maximum discriminatory power is shown in **Table 2**; see **Supplementary Information File 1**, **Table S3** for detail. These top 10 genes showed highest performance with an accuracy > 85%, MCC > 0.75, and AUROC > 0.85. We also evaluate the performance of these top 10 genes using 10-fold cross-validation to understand their robustness as shown in **Table S4** (**Supplementary Information File 1**). We further selected 5 genes out of 10 genes, which exhibit the maximum performance. These genes are *FCN3, CLEC1B, CLEC4M, PRC1*, and *PBK*; models based on these genes have accuracy more than 90% with AUROC > 0.95. In addition, the performance is also evaluated on the external validation datasets. The performance of the method was same on the training dataset but decreases on the external validation for few genes/features (see **Table S5**, **Supplementary Information File 1**).

**Table 2:** Top 10 genes based on the simple threshold-based approach.

**Gene symbol**	**Thresh**	**Sens (%)**	**Spec (%)**	**Acc (%)**	**MCC**	**AUROC**	**Mean in HCC**	**Mean in normal**	**Mean diff**
***FCN2***	9.78	97.76	99.59	98.63	0.97	0.98	5.76	10.89	–5.13
***CLEC4M***	7.59	97.01	98.77	97.85	0.96	0.98	4.32	9.37	–5.06
***FCN3***	10.76	95.15	99.18	97.06	0.94	0.97	7.87	12.32	–4.45
***CLEC1B***	9.46	95.52	97.94	96.67	0.93	0.97	5.96	11.38	–5.42
***CFP***	8.14	96.64	94.24	95.50	0.91	0.96	6.15	8.63	–2.48
***CRHBP***	8.69	92.54	96.71	94.52	0.89	0.95	6.35	10.30	–3.95
***PRC1***	7.76	91.42	97.12	94.13	0.88	0.94	10.03	6.35	3.68
***PBK***	6.03	91.04	93.42	92.17	0.84	0.93	8.65	4.41	4.24
***DTL***	6.71	85.82	94.65	90.02	0.80	0.91	8.72	5.20	3.52
***IGSF3***	6.93	81.34	91.77	86.30	0.73	0.88	8.10	6.08	2.01

Sens, Sensitivity; Spec, Specificity; Acc, Accuracy; MCC, Mathews Correlation Coefficient; AUROC, Area under Receiver operator curve; Thresh, Threshold; Mean diff, Mean in HCC–Mean in normal.

#### Multiple-Genes Based Prediction Models

**Figure 3:**
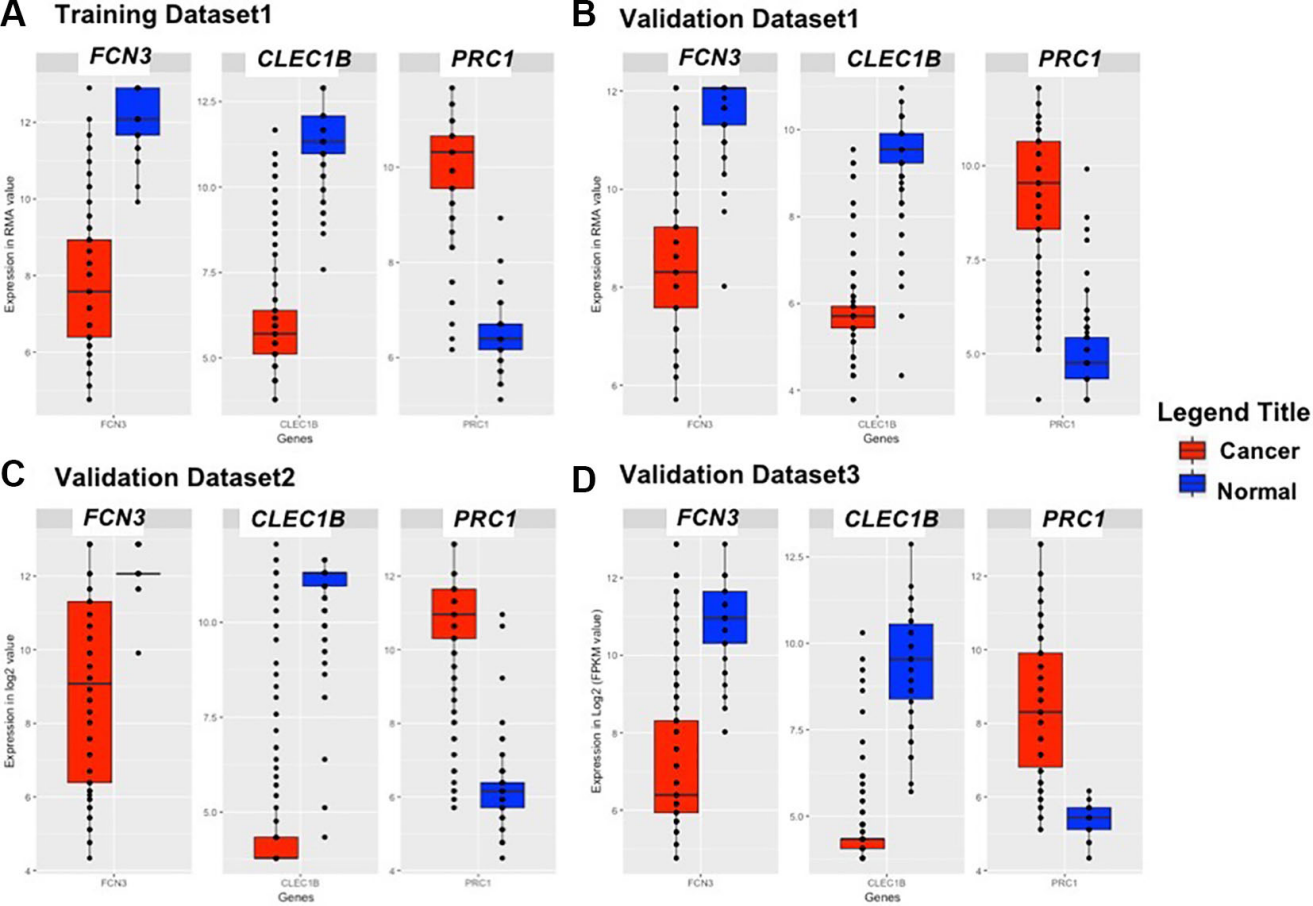
Boxplot representing the expression pattern of three-genes panel-based HCC biomarker in the **(A)** Training Dataset, **(B)** Validation Dataset 1, **(C)** Validation Dataset 2, **(D)** Validation Dataset 3.

We identified the top five genes based on single gene-based prediction models, as described above. Further, we developed machine learning techniques-based classification models using these top five genes. We called these models as multiple-genes based prediction models as they take multiple genes as input. These models were evaluated on the training as well as validation datasets using internal and external cross-validation. The performance of these models on training as well as on three validation datasets is shown in **Table 3**. As shown in **Table 3**, we got AUROC approximately 0.98 on training as well as on the validation datasets. We further reduced one gene from selected set of five genes using feature reduction technique as described in *Materials and Methods* and obtained a set of four genes (*FCN3, CLEC1B, PRC1, PBK*). Subsequently, machine learning prediction models developed based on them classified HCC and non-tumor samples with accuracy more than 95% with AUROC in the range of 0.97–0.99 on both training and three independent validation datasets as shown in **Table S6** (**Supplementary Information File 1**). Results from this analysis show that we got nearly same performance using four genes-based biomarkers as we got in case of five genes-based biomarkers. Thus, reduction of one feature (five to four) does not affect the performance of our multiple-gene based prediction method. We further reduced features using feature reduction technique and got a set of three genes that contains *FCN3, CLEC1B,* and *PRC1*. Prediction models based on three genes-biomarker got accuracy 95–98% with AUROC in the range of 0.96–0.99 on training as well as independent validation datasets as shown in **Table 4**. The expression pattern of these three genes among samples of training dataset and three external validation datasets is depicted in **Figure 3**. We also tried two gene biomarkers, but there is substantial reduction in the performance on validation datasets. Thus, our final model is developed using a biomarker panel of three genes that include *FCN3, CLEC1B,* and *PRC1.* We considered three-genes based biomarker as the final model because the number of genes is limited. Hence, it is easy to implement in real life as well as economical.

**Table 3:** Performance of five genes (*FCN3*, *CLEC4M*, *CLEC1B*, *PRC1*, *PBK*) based models on training and validation datasets implementing various machine learning techniques.

**Classifier**	**Sens (%)**	**Spec (%)**	**Acc** **(%)**	**MCC**	**AUROC with 95% CI**	**Sens (%)**	**Spec (%)**	**Acc** **(%)**	**MCC**	**AUROC** **with 95% CI**
	**Training Dataset**					**Validation Dataset1**				
**ETREES**	97.39	98.35	97.85	0.96	0.99 (0.99-1)	97.78	94.09	95.96	0.92	0.98 (0.97-0.99)
**NB**	97.76	99.18	98.43	0.97	0.99 (0.99-1)	97.33	95.45	96.40	0.93	0.98 (0.97-0.99)
**KNN**	97.39	98.77	98.04	0.96	0.99 (0.99-1)	96.89	96.82	96.85	0.94	0.98 (0.97-0.99)
**RF**	97.01	97.94	97.46	0.95	0.99 (0.99-1)	97.33	94.55	95.96	0.92	0.98 (0.97-0.99)
**LR**	97.76	99.59	98.63	0.97	0.99 (0.99-1)	95.56	97.27	96.40	0.93	0.99 (0.98-0.99)
**SVC**	97.01	100	98.43	0.97	0.99 (0.99-1)	96.89	95.00	95.96	0.92	0.99 (0.98-0.99)
	**Validation Dataset2**					**Validation Dataset3**				
**ETREES**	95	97.41	96.07	0.92	0.98 (0.97-0.99)	97.86	96	97.64	0.89	0.99 (0.98-0.99)
**NB**	94.58	98.45	96.3	0.93	0.98 (0.96-0.99)	98.13	92	97.41	0.88	0.98 (0.98-0.99)
**KNN**	92.92	98.45	95.38	0.91	0.97 (0.96-0.99)	97.86	94	97.41	0.88	0.99 (0.98-0.99)
**RF**	96.67	93.26	95.15	0.9	0.98 (0.97-0.99)	98.4	90	97.41	0.88	0.99 (0.98-0.99)
**LR**	93.75	98.45	95.84	0.92	0.98 (0.97-0.99)	97.59	98	97.64	0.90	0.99 (0.98-0.99)
**SVC-RBF**	93.33	98.45	95.61	0.91	0.98 (0.97-0.99)	97.33	98	97.41	0.89	0.99 (0.98-0.99)

ETREES, Extra Trees Classifier; NB, Naive Bayes; KNN, K Neighbors Classifier; RF, Random Forest; LR, Logistic Regression; SVC-RBF, Support Vector Machine with RBF-kernel; Sens, Sensitivity; Spec, Specificity; Acc, Accuracy; MCC, Mathews Correlation Coefficient; AUROC, Area under Receiver operator curve.

**Table 4:** Performance of three-genes HCC biomarker-A (*FCN3*, *CLEC1B*, *PRC1*) based models on training and validation datasets implementing various machine learning techniques.

**Classifier**	**Sens (%)**	**Spec (%)**	**Acc** **(%)**	**MCC**	**AUROC with 95% CI**	**Sens (%)**	**Spec (%)**	**Acc** **(%)**	**MCC**	**AUROC with 95% CI**
	**Training Dataset**					**Validation Dataset1**				
**ETREES**	96.64	97.94	97.26	0.95	0.99 (0.98-0.99)	94.67	95.91	95.28	0.91	0.97 (0.96-0.99)
**NB**	97.39	99.18	98.24	0.96	0.99 (0.99-1.0)	96.00	95.91	95.96	0.92	0.98 (0.97-0.99)
**KNN**	97.76	98.77	98.24	0.96	0.99 (0.99-1.0)	93.78	97.73	95.73	0.92	0.97 (0.96-0.99)
**RF**	97.01	97.53	97.26	0.95	0.99 (0.99-1.0)	94.67	96.36	95.51	0.91	0.97 (0.96-0.99)
**LR**	93.28	100	96.48	0.93	0.99 (0.99-1.0)	92.89	97.73	95.28	0.91	0.98 (0.97-0.99)
**SVC-RBF**	94.03	100	96.87	0.94	0.99 (0.98-0.99)	96.00	96.82	96.40	0.93	0.98 (0.97-0.99)
	**Validation Dataset2**					**Validation Dataset3**				
**ETREES**	93.75	96.37	94.92	0.90	0.98 (0.97-0.99)	95.72	98	95.99	0.84	0.99 (0.98-0.99)
**NB**	94.58	98.45	96.3	0.93	0.98 (0.97-0.99)	98.13	82	96.23	0.82	0.96 (0.95-0.98)
**KNN**	95.83	97.93	96.77	0.94	0.98 (0.97-0.99)	97.59	96	97.41	0.88	0.99 (0.98-0.99)
**RF**	95.42	94.3	94.92	0.90	0.98 (0.97-0.99)	95.45	96	95.52	0.82	0.98 (0.97-0.99)
**LR**	95.42	98.45	96.77	0.94	0.99 (0.98-0.99)	97.33	98	97.41	0.89	0.99 (0.98-0.99)
**SVC-RBF**	93.33	97.93	95.38	0.91	0.98 (0.97-0.99)	96.79	98	96.93	0.87	0.99 (0.98-0.99)

ETREES, Extra Trees Classifier; NB, Naive Bayes; KNN, K Neighbors Classifier; RF, Random Forest; LR, Logistic Regression; SVC-RBF, Support Vector Machine with RBF kernel; Sens, Sensitivity; Spec, Specificity; Acc, Accuracy; MCC, Mathews Correlation Coefficient; AUROC, Area under Receiver operator curve.

#### Validation of Models on Blood Samples

In this study, models have been developed on tissue samples, which is complex and difficult to implement for routine testing. The question arises whether this model can also be used to discriminate the samples achieved from non-invasive techniques. Thus, we assessed the performance of our final model on PBMCs/blood samples of GSE36076. These signature genes correctly predicted 90% of both HCC and healthy samples with ROC in the range of 0.91–0.96 and MCC 0.80–0.82. Complete results of prediction models are tabulated in **Table 5**. This demonstrates that our three genes-based models have the ability to discriminate HCC and healthy blood samples with reasonably high accuracy.

**Table 5:** Performance of three-genes HCC biomarker-A (*FCN3*, *CLEC1B*, *PRC1*) based models on training and validation datasets 4 (containing blood samples, i.e., PBMCs) implementing various machine learning techniques.

**Classifier**	**Sens (%)**	**Spec (%)**	**Acc** **(%)**	**MCC**	**AUROC with 95% CI**	**Sens (%)**	**Spec (%)**	**Acc** **(%)**	**MCC**	**AUROC with 95% CI**
	**Training Dataset**					**Validation Dataset4**				
**ETREES**	94.78	99.18	96.87	0.94	0.99 (0.979-0.998)	100	80	90	0.82	0.93 (0.854-1.0)
**NB**	97.39	99.18	98.24	0.96	0.99 (0.989-1.0)	90	90	90	0.80	0.95 (0.81-1.0)
**KNN**	97.01	99.59	98.24	0.97	0.99 (0.986-1.0)	90	90	90	0.80	0.96 (0.878-1.0)
**RF**	95.52	99.59	97.46	0.95	0.99 (0.991-1.0)	100	80	90	0.82	0.93 (0.81-1.0)
**LR**	96.64	100	98.24	0.97	0.99 (0.992-1.0)	90	90	90	0.80	0.96 (0.877-1.0)
**SVC**	95.15	99.18	97.06	0.94	0.99 (0.988-0.999)	90	90	90	0.80	0.91 (0.744-1.0)

ETREES, Extra Trees Classifier; NB, Naive Bayes; KNN, K Neighbors Classifier; RF, Random Forest; LR, Logistic Regression; SVC-RBF, Support Vector Machine with RBF kernel; Sens, Sensitivity; Spec, Specificity; Acc, Accuracy; MCC, Mathews Correlation Coefficient; AUROC, Area under Receiver operator curve.

### Protein-Based Biomarkers

In the past, proteins have been identified as diagnostic biomarkers for HCC. These protein biomarkers are AFP+GPC3 and AFP+GPC3+CK19 (*KRT19*) (Lou et al., 2017; Ocker, 2018). As we do not have their protein expression for these patients’ samples, we employed only their gene expression values. Models based on the gene expression of *AFP+GPC3+KRT19* classified HCC and normal samples of training dataset with an accuracy 67–75%. While this model attained accuracy of 69–77%, 51–87%, and 50–74% on external validation dataset1, dataset2 and dataset3, respectively, as shown in **Table S7** (**Supplementary Information File 1**). Further, the prediction models based on the gene expression of *AFP+GPC3* have improved performance on training dataset with an accuracy of 70–77%, but lower performance on all three validation datasets as given in **Table S8** (**Supplementary Information File 1**).

### Survival Analysis to Determine the Prognostic Potential of “Three-Genes HCC Biomarker”

#### Univariate Survival Analysis for Three-Genes HCC Biomarker

**Figure 4:**
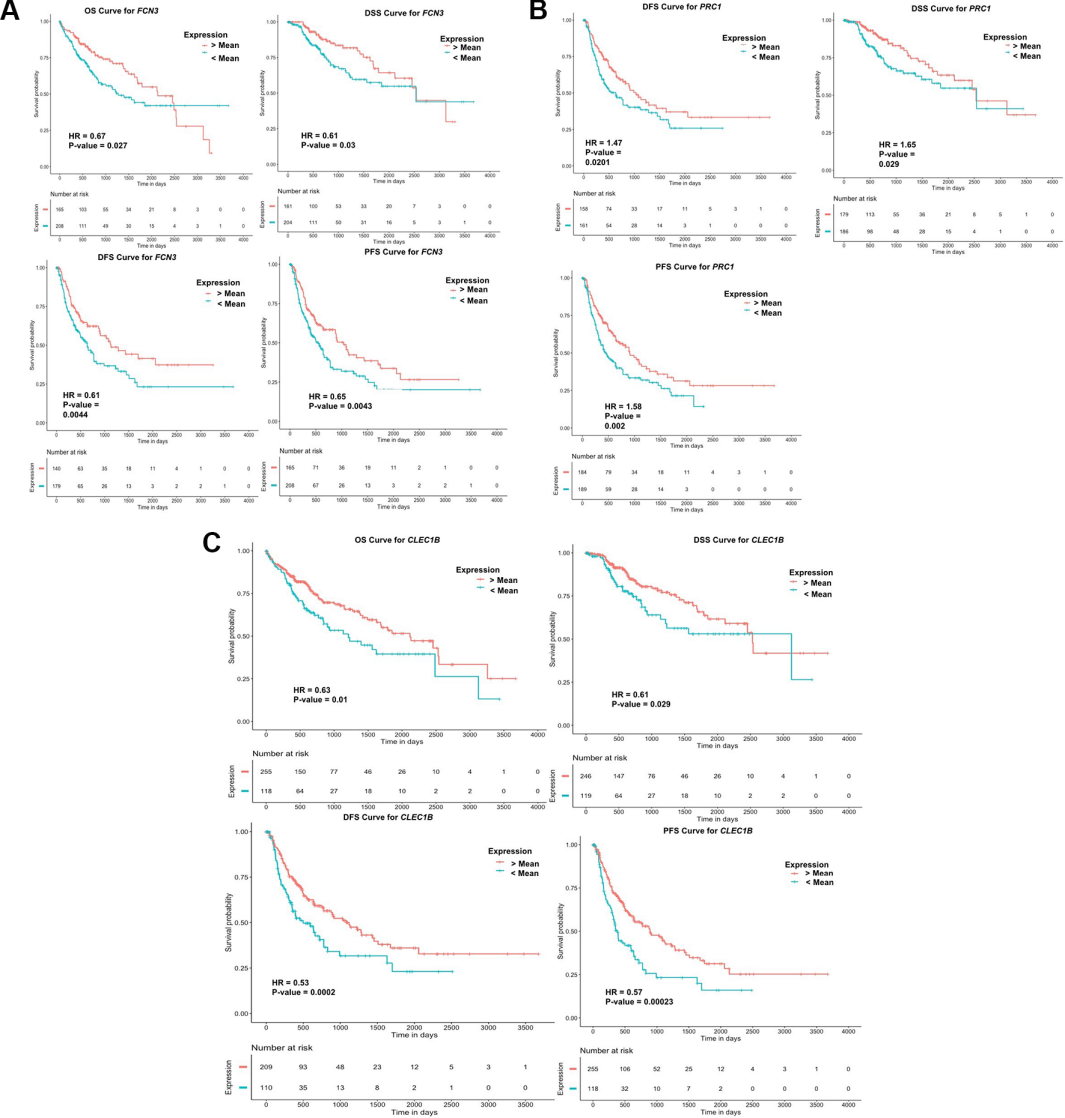
Kaplan Meier survival curves for the risk estimation of HCC patient in TCGA cohort based on the RNA expression of **(A)** *FCN3*, **(B)** *PRC1*, and **(C)** *CLEC1B*.

**Figure 5:**
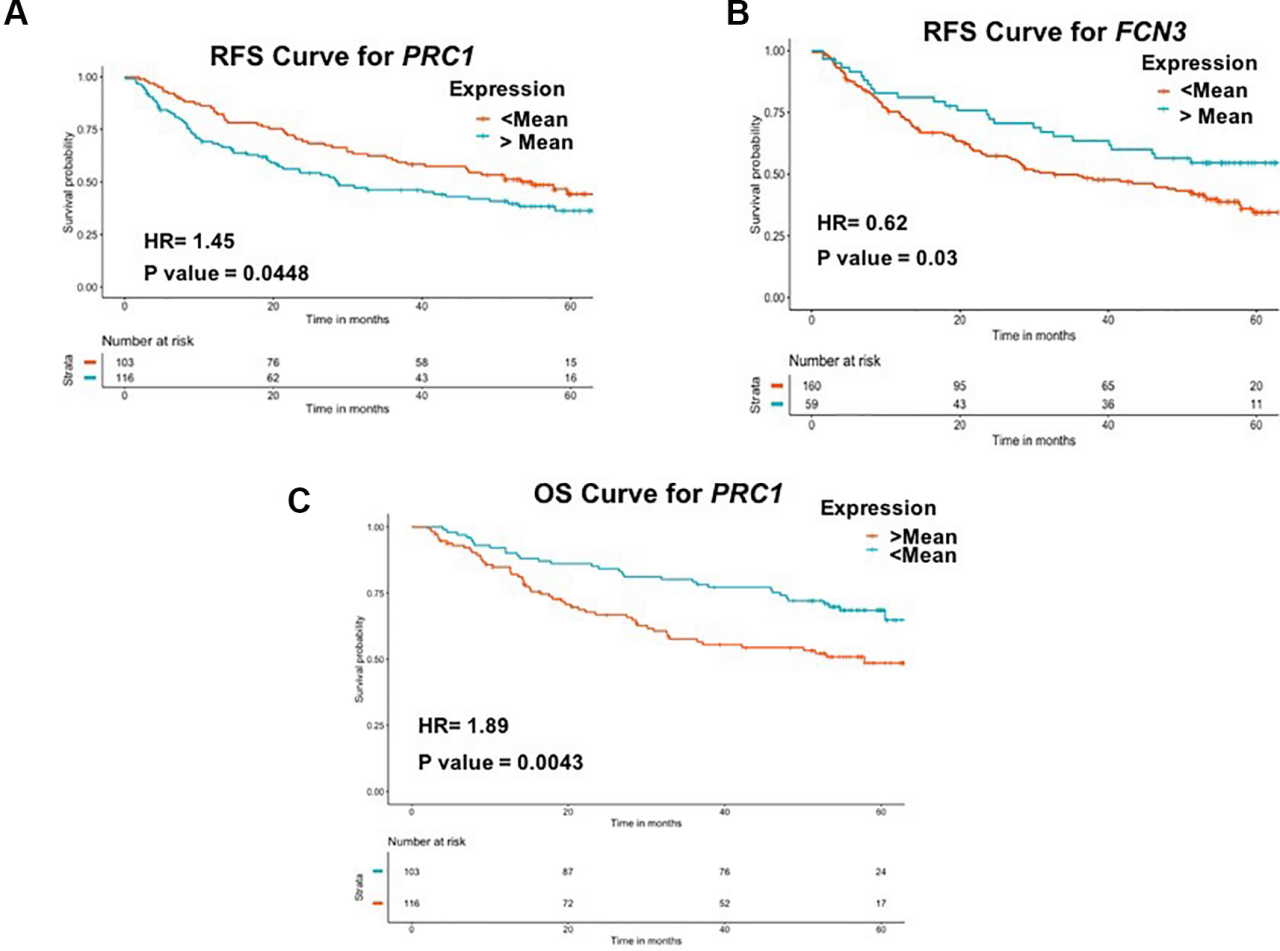
Kaplan Meier survival curves for the risk estimation of HCC patient in GSE14520 cohort based on the RNA expression of **(A)** RFS for *PRC1*, **(B)** RFS for *FCN3*, and **(C)** OS for *PRC1*.

To examine the prognostic potential of the “three-genes HCC biomarker,” the univariate survival analysis was performed on TCGA-LIHC and GSE14520 cohorts. The samples were partitioned into low-risk and high-risk groups. Interestingly, all three genes of “three-genes HCC biomarker-A” are significantly associated with the survival of HCC patients. For instance, higher expression (greater than mean) of *CLEC1B* and *FCN3* is significantly associated with good outcome of the patients, i.e. OS, DSS, DFS, and PFS; while the overexpression of *PRC1* is significantly associated with poor survival including DSS, DFS, or RFS and PFS of HCC patients for TCGA-LIHC dataset as shown in **Figure 4**. In the GSE14520 dataset, higher expression of *PRC1* is significantly associated with the poor outcome of patients, i.e., OS and DFS or RFS, while the higher expression of *FCN3* is significantly associated with the better outcome of HCC patients as depicted in **Figure 5**. Complete results of survival analysis with HR (Hazard Ratio), with 95% CI and p-value, are presented in **Table S9** (**Supplementary Information File 1**).

#### Univariate Survival Analysis for Clinical Features

The clinical characteristics of the patients like age, gender, tumor size, and stage are considered as important prognostic indicators for the survival of the patients in different malignancies including HCC (Best et al., 2016; Liu et al., 2018a; Wu et al., 2018; Yang et al., 2019). As the tumor size information is not present in one of the cohorts, therefore, we performed univariate survival analysis using only age, gender, and tumor stage of the patients. This analysis shows that tumor stage is an important clinical factor with prognostic potential that significantly stratified high-risk and low-risk groups of patients in both cohorts, i.e., TCGA-LIHC and GSE14520. For instance, stage individually significantly (p-value <0.0001) stratified risk groups for OS, RFS with HR = 1.73 and HR = 1.65 of TCGA cohorts and with HR = 2.29 and HR = 1.79 of GSE14520 cohort, respectively (**Table S10**, **Supplementary Information File 1**). While the gender and age of patients do not possess high prognostic potential, as shown in **Table S10** (**Supplementary Information File 1**).

#### Multivariate Survival Analysis

Eventually, the multivariate analysis is performed to assess the independent impact of clinical characteristics and three genes of our signature biomarker that are determined as significant prognostic variables by univariate analysis. From this analysis, tumor stage is identified as the sole independent prognostic factor associated with the survival of HCC patients that significantly (with p-value <0.01) stratified high-risk and low-risk groups of both TCGA-LIHC and GSE1450 cohorts as presented in **Figures S4–S6** (**Supplementary Information File 2**).

### Web Server

To facilitates the scientific community working in the area of liver cancer research, we developed “HCCpred” (Prediction Server for Hepatocellular Carcinoma). In HCCpred, we execute mainly two modules: Prediction Module and Analysis Module based on robust five-genes, four-genes. and three-genes HCC biomarkers and 26 Core genes of HCC identified in the present study for the prediction and analysis of samples from the RNA-expression data. The prediction module permits the users to predict the disease status, i.e., cancerous or normal using RNA expression values of a subset of genes using *in silico* prediction models based on robust five-genes, four-genes, and three-genes HCC biomarkers identified in the present study. Here, the user is required to submit RMA (for Affymetrix), A-value (for Agilent), Log2 value (for Illumina), or FPKM (High throughput RNA-seq data) for a subset of genes or biomarkers. The output result displays a list for patient samples and corresponding predicted status of samples. Moreover, the user can select among the models, i.e., ETREES-based or SVC-RBF based model. Further, the Analysis Module permits the user to analyze the expression pattern of any of the top 10 ranked genes to check whether it is upregulated or downregulated in comparison to HCC samples based on the samples of the current study. This webserver is freely accessible at http://webs.iiitd.edu.in/raghava/hccpred/.

## Discussion

HCC is a type of tumor that is associated with the poor prognosis and a high mortality rate among the most common cancer types (Siegel et al., 2019). High recurrence rate and low rate of early detection results in poor prognosis. Accurate diagnosis of HCC may provide the opportunity for appropriate treatment, including traditional available treatment like liver transplantation resection, etc. Although the AFP and DCP proteins are well-established markers for the diagnosis of HCC, their sensitivity and specificity are not optimum (Sauzay et al., 2016). Therefore, the development of a novel robust diagnostic and prognostic biomarker for HCC is needed as it can assist in the existing clinical management of tumor. Towards this, our current report is an attempt to scrutinize a robust transcriptomic biomarker for HCC diagnosis. Briefly, in this study, we provide a novel large-scale analysis-based approach to identify a robust gene expression-based candidate diagnostic biomarker for HCC derived from multiple transcriptomic profiles/datasets across a variety of platforms obtained from GEO and TCGA. This metadata integration approach employed to elucidate “core HCC DEGs” subset followed by a class prediction by implementing various machine learning algorithms. Eventually, validation on external independent datasets led us to the identification of multiple-genes based robust biomarkers for HCC.

Here, firstly, we have identified 26 genes named as “Core DEGs for HCC” that are uniformly differentially expressed among 80% of datasets. We have considered only these genes for downstream machine learning analysis. In an urge to identify a manageable subset with the minimum number of genes from this list that have a high discriminatory power, we further identified three genes signature-set containing *CLEC1B, FCN3*, and *PRC1*. This “three-genes based HCC biomarker” has predictive accuracy of 95–98% and AUROC 0.96–0.99 on the training and all three independent validation datasets. We further hypothesized that this biomarker gene set might be proved as quite an effective non-invasive diagnostic biomarker for HCC. Therefore, eventually, we validated their discriminatory performance on 20 PBMCs samples (GSE36076) extracted from 10 HCC and 10 healthy individuals. As anticipated, this biomarker set correctly classified 90% of the samples with AUROC in the range of 0.91–0.96. Besides, we also developed the prediction models based on the gene expression of already well-established protein biomarkers of HCC in the literature, i.e., *AFP+GPC3* and *AFP+GPC3+KRT19* (Lou et al., 2017). The prediction models based on *AFP+GPC3+KRT19* discriminate samples of training dataset with an accuracy of 67–75% and 69–77% of validation dataset1, 55–87% of validation dataset2, and 50–74% of validation dataset3, while the models based on *AFP+GPC3* have quite lower performance on validation datasets. Further, we speculate that “three-genes HCC biomarker” can be explored as an effective novel protein based non-invasive biomarker as they have very good predictive power to distinguish HCC and non-tumor samples at gene expression level from the tissue and PBMC samples. Moreover, the product of *FCN3* gene is released in the serum and bile (Akaiwa et al., 1999; Brown et al., 2015; Pan et al., 2015; Tizzot et al., 2018); thus, this may serve as non-invasive biomarkers for diagnosis of HCC. Furthermore, recently, it has been reported that the protein product of two of the three genes from three-genes HCC biomarker, i.e., *PRC1* and *FCN3*, is also associated with HCC diagnosis and prognosis independently (Liu et al., 2018b; Shen et al., 2018). Hence, we anticipate that the three-genes signature might prove to be a good diagnostic and prognostic marker for HCC at the protein level as well. There is still a need for the validation of the protein product of these genes on a large scale of samples to confirm this hypothesis and their clinical utility.

Interestingly, the robust “three-genes HCC biomarker” contains *FCN3, PRC1,* and *CLEC1B,* has very high diagnostic ability, and also possesses prognostic potential, i.e., they are significantly associated with survival of HCC patients as determined by univariate analysis. For instance, higher expression of *CLEC1B* and *FCN3* significantly associated with the good outcome of HCC patients in TCGA-LIHC cohort; while higher expression of *PRC1* is significantly associated with the poor outcome of HCC patients in both TCGA-LIHC and GSE14520 cohorts. Besides, the role of *CLEC1B* and *PRC1* was previously also revealed in the diagnosis and prognosis of HCC (Chen et al., 2016; Chan et al., 2018; Hu et al., 2018; Kaur et al., 2019). Further, univariate analysis employing clinical factors of patients found that tumor stage of patients can act as a strong prognostic factor in the various types of survival, i.e., OS, RFS/DFS, PFS, and DSS of patients. Eventually, the multivariate survival analysis revealed the tumor stage as a sole independent prognostic factor, which was also corroborated with the previous literature (Aino et al., 2014; Wang and Li, 2019). The correct tumor stage identification is quite a tedious and challenging task in comparison to the quantification of the expression of genes.

In the past, a concern raised by Kaplan et al. is that despite the number of advantages of big studies, large sample size can also magnify the bias associated with an error resulting from sampling or study design (Kaplan et al., 2014). Thus, to reduce the overestimation of inferences from the results of large cohorts, we have included both types of cohorts, i.e., large cohort (sample size >50) and small cohort (sample size <50). We hypothesized that these results might be more reliable and applicable. Additionally, it might be practically more useful in real life, where, usually, small cohorts are available with maximum clinical parameters. Therefore, to ensure that cohort’s size does not affect the results derived from the overall study, results should be validated on a small cohort as well. Towards this, we have also validated models built on the training dataset on three large cohorts of external validation dataset and one small cohort (contains 20 blood samples). Thus, these results indicate that there is no overestimation of inferences from the results of cohorts used in the study.

Taken together, we have established a robust three-gene HCC diagnostic biomarker with reasonable performance and possesses both diagnostic and prognostic potential. A meta-data integration pipeline is employed for the identification of a robust biomarker using machine learning techniques, which can work across different platforms. Further, this pipeline can also be used for the analysis of any other cancer type. Although more and more research is under the development of novel biomarkers, further work will be required to implement the clinical utilization of identified biomarker to meet real-world demand. We are anticipating that identifying novel cost-efficient biomarker using predictive technology for the detection of HCC will be promising.

## Conclusions

This study identified and validated a highly accurate three-genes HCC biomarker for discriminating HCC and non-tumorous samples; it also possesses a significant prognostic potential that may facilitate more accurate early diagnosis and risk stratification upon validation in prospective clinical trials. Reasonable performance on the validation dataset of PBMCs samples indicates their non-invasive utility. Moreover, the protein product of *FCN3* is released in the serum and bile. Thus, this may serve as non-invasive protein diagnostic biomarkers. Large-scale non-invasive cohorts are required to confirm their non-invasive clinical utility. Additionally, the uniform overexpression pattern of *PRC1* among numerous HCC samples suggests it as a novel potential therapeutic target for HCC.

## Data Availability Statement

We have taken the Gene-expression data from the public repositories, i.e., GEO (https://www.ncbi.nlm.nih.gov/geo/) and GDC data portal (https://portal.gdc.cancer.gov/). 

## Author Contributions

HK collected the data and created the datasets. HK developed classification algorithms. HK and AD implemented algorithms. HK and AD performed the survival analysis. HK and AD created the back-end server and front-end user interface. HK and GR analyzed the results. HK, RK, and AD wrote the manuscript. GR conceived and coordinated the project, helped in the interpretation and analysis of data, refined the drafted manuscript, and gave complete supervision to the project. All of the authors have read and approved the final manuscript.

## Funding

This research was funded by J. C. Bose National Fellowship (with Grant No. SRP076), Department of Science and Technology (DST), India.

## Conflict of Interest

The authors declare that the research was conducted in the absence of any commercial or financial relationships that could be construed as a potential conflict of interest.

## Abbreviations

AUROC, Area under the Receiver Operating Characteristic curve; ETREES, Extra Trees Classifier; SVC-RBF, Support Vector Machine with RBF kernel; TCGA, The Cancer Genome Atlas; KNN, K Neighbors Classifier; HCC, Hepatocellular Carcinoma; MCC, Matthew’s correlation coefficient; LR, Logistic Regression; NB, Naive Bayes; RF, Random Forest; PBMCs, Peripheral Blood Mononuclear Cells

## Supplementary Material

The Supplementary Material for this article can be found online at: https://www.frontiersin.org/articles/10.3389/fgene.2019.01306/full#supplementary-material

Supplementary Figure S1 Distribution of Significantly DEG (Differentially Expressed Genes) among various datasets with Bonferroni adjusted p-value < 0.01.

Supplementary Figure S2 Heatmap representing the expression pattern of “Core genes of HCC” in different datasets.

Supplementary Figure S3 Gene Enrichment analysis of 26 genes or “Core genes of HCC”.

Supplementary Figure S4 Multivariate analysis of clinical characteristics and three genes of HCC Biomarker on TCGA cohort for **(A)** OS, **(B)** RFS/DFS.

Supplementary Figure S5 Multivariate analysis of clinical characteristics and three genes of HCC Biomarker on TCGA cohort for **(A)** DSS, **(B)** PFS.

Supplementary Figure S6 Multivariate analysis of clinical characteristics and three genes of HCC Biomarker on GSE14520 cohort for **(A)** OS, **(B)** RFS/DFS.
