# Supplementary figures and images for: Identification of Platform-Independent Diagnostic Biomarker Panel for Hepatocellular Carcinoma Using Large-Scale Transcriptomics Data
Source: Front Genet. 2020 Jan 10;10:1306. doi: 10.3389/fgene.2019.01306 (PMC6967266; doi:10.3389/fgene.2019.01306)

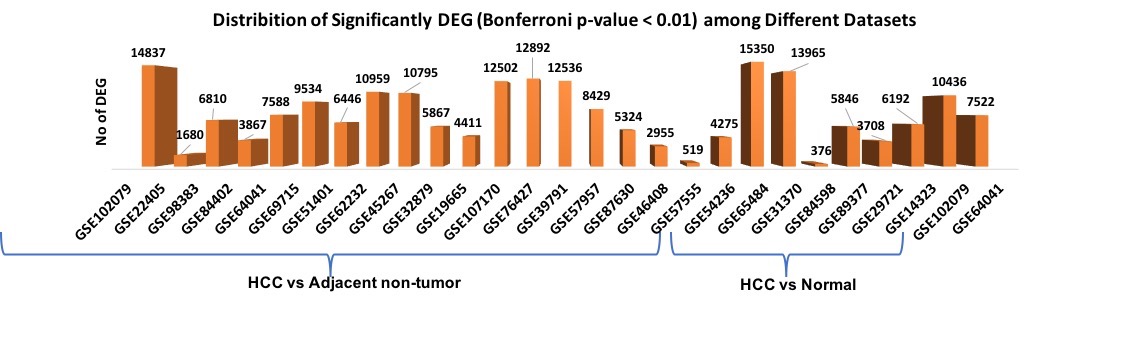

Supplement: Supplementary Figure S1 — Distribution of Significantly DEG (Differentially Expressed Genes) among various datasets with Bonferroni adjusted p-value < 0.01. [file Image_1.jpeg]

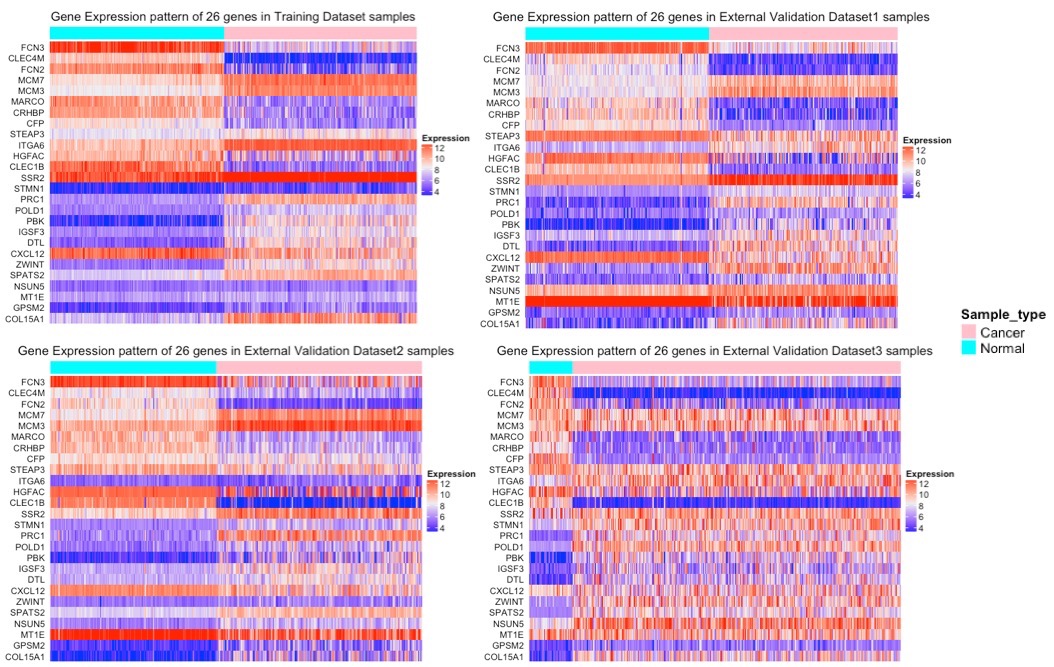

Supplement: Supplementary Figure S2 — Heatmap representing the expression pattern of “Core genes of HCC” in different datasets. [file Image_2.jpeg]

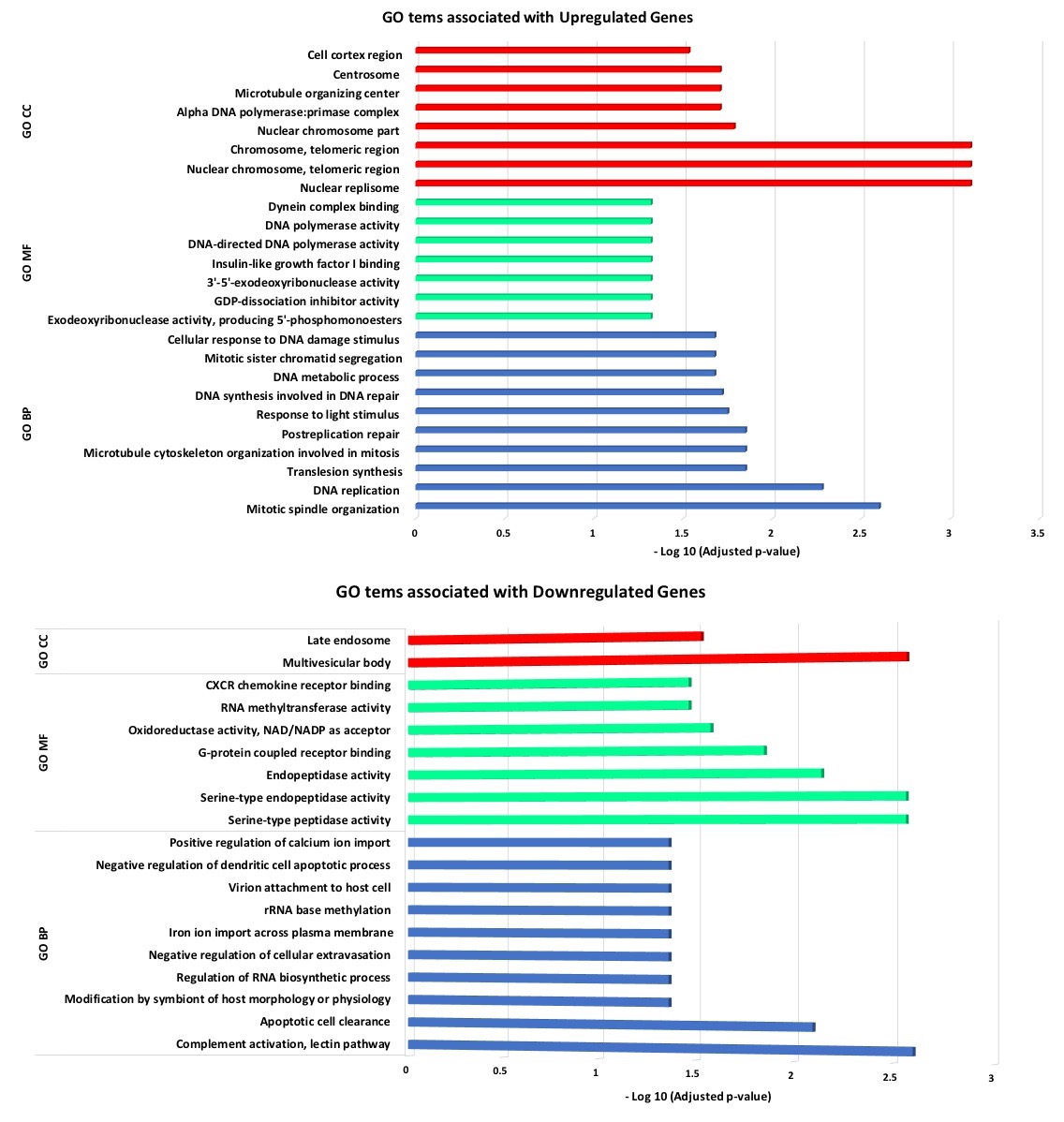

Supplement: Supplementary Figure S3 — Gene Enrichment analysis of 26 genes or “Core genes of HCC”. [file Image_3.jpeg]

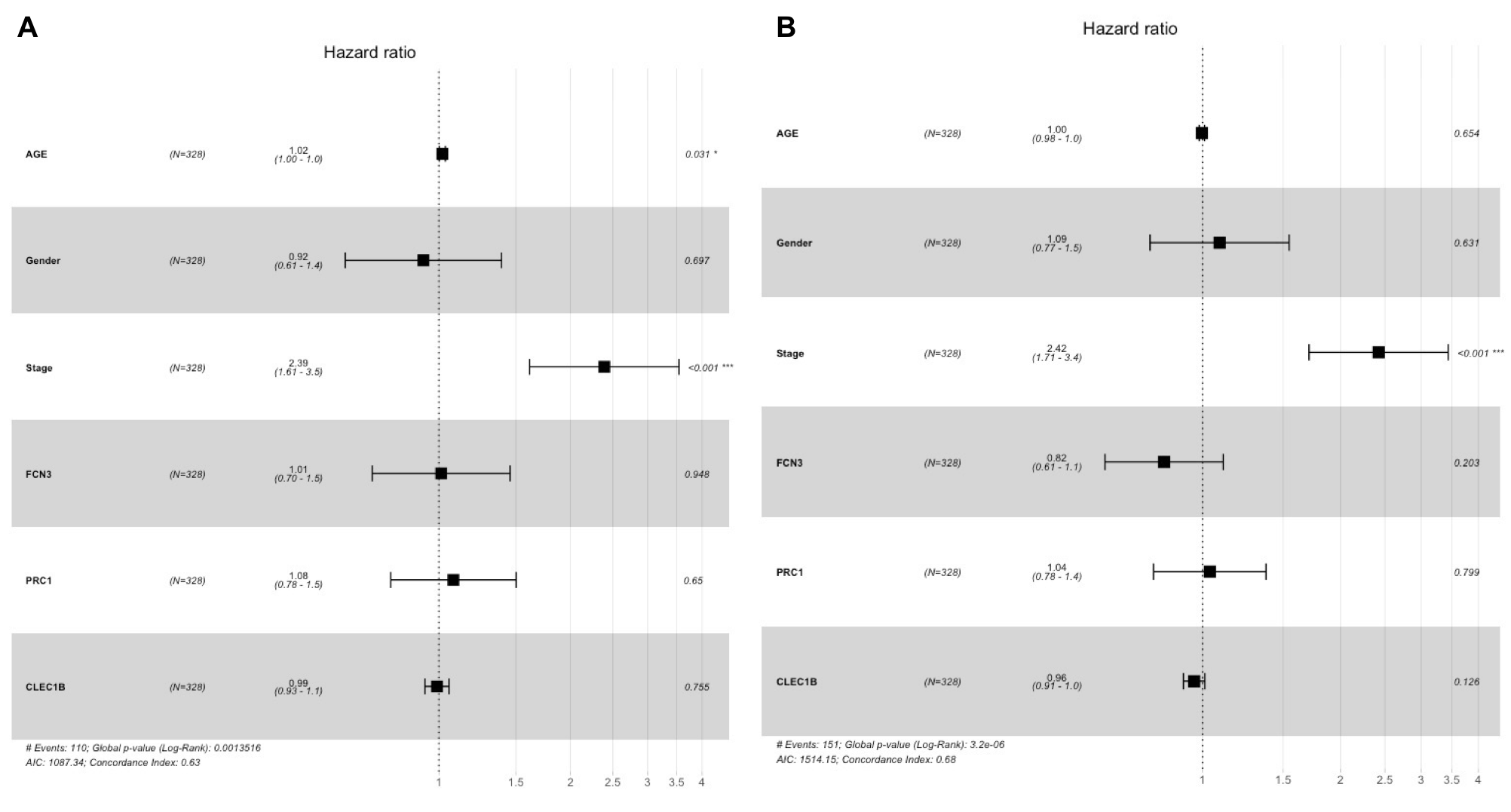

Supplement: Supplementary Figure S4 — Multivariate analysis of clinical characteristics and three genes of HCC Biomarker on TCGA cohort for (A) OS, (B) RFS/DFS. [file Image_4.png]

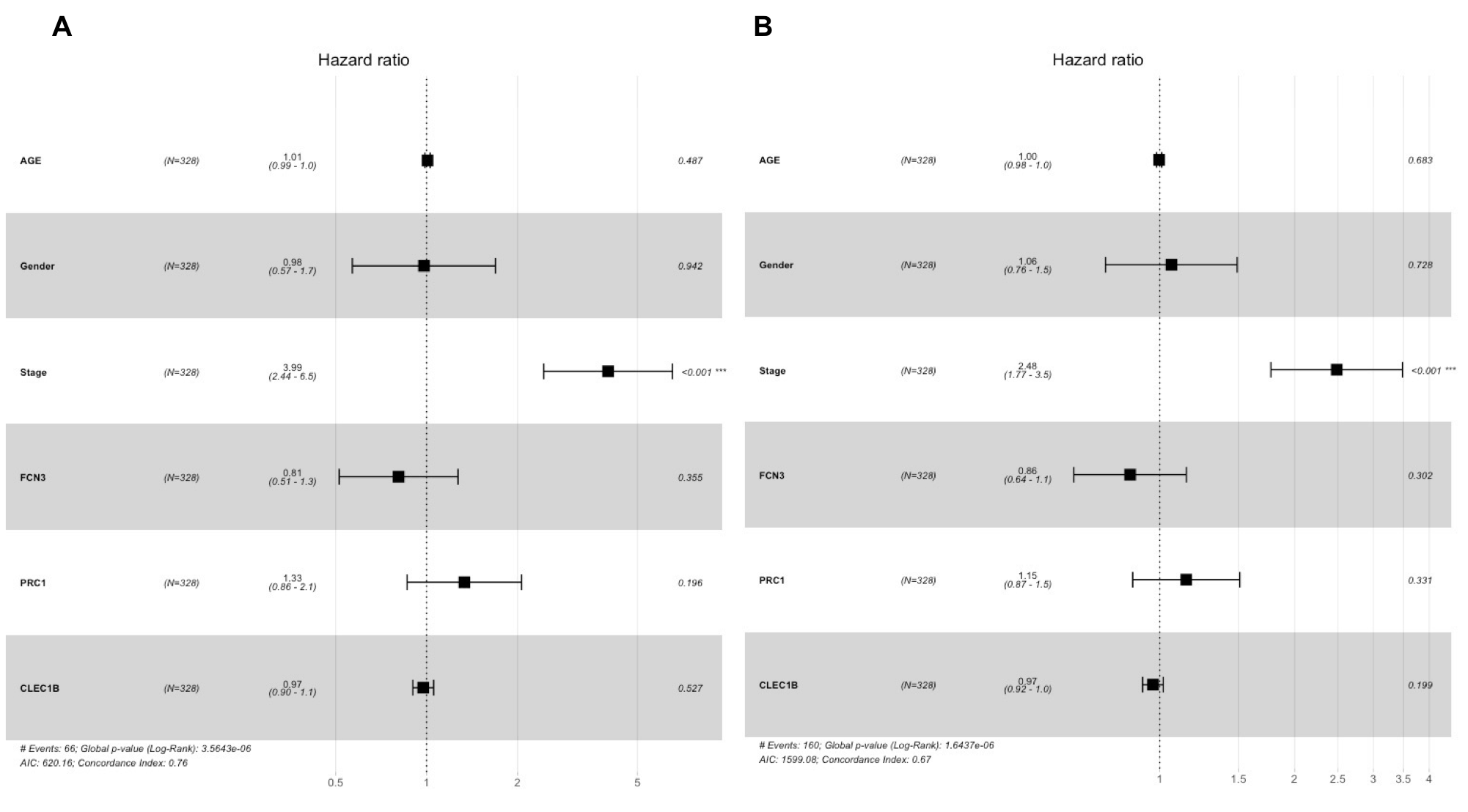

Supplement: Supplementary Figure S5 — Multivariate analysis of clinical characteristics and three genes of HCC Biomarker on TCGA cohort for (A) DSS, (B) PFS. [file Image_5.png]

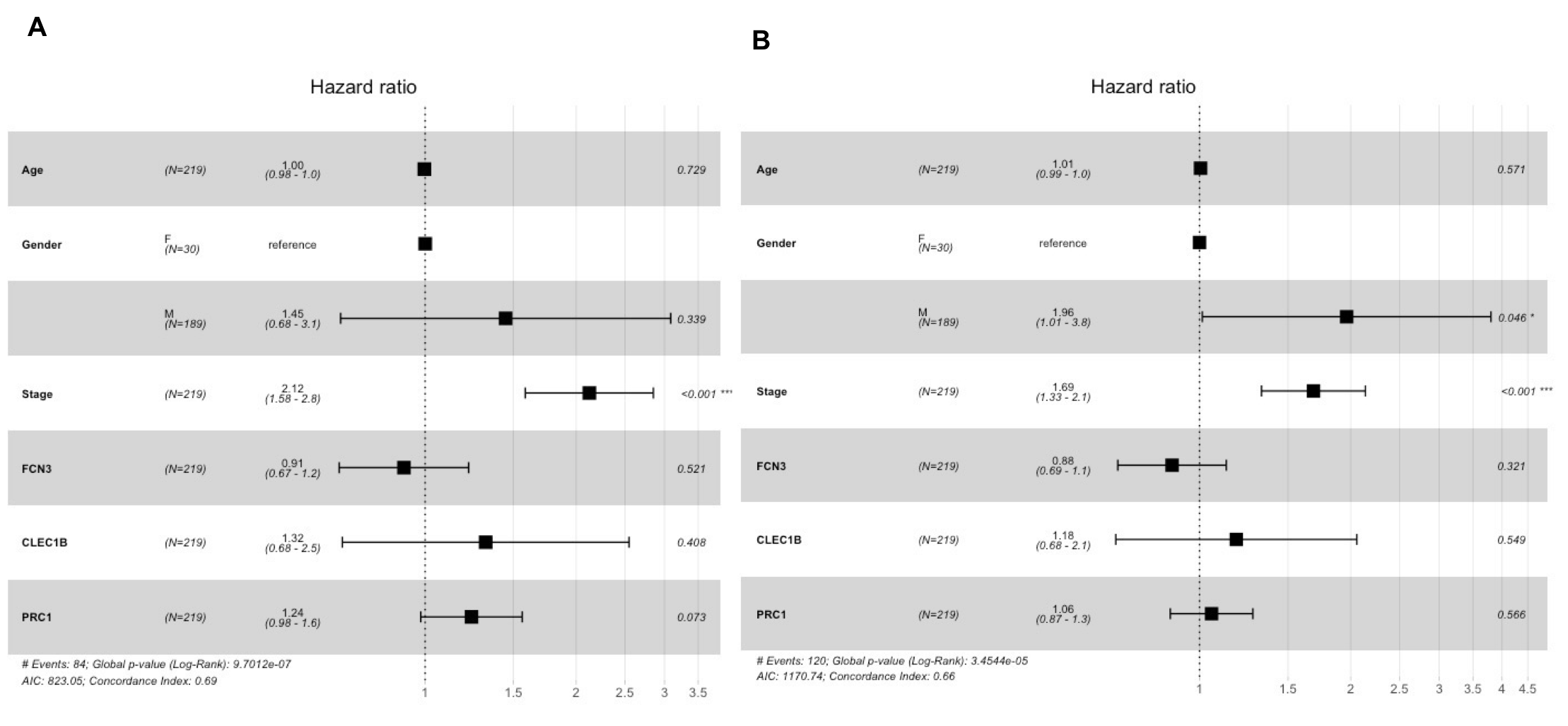

Supplement: Supplementary Figure S6 — Multivariate analysis of clinical characteristics and three genes of HCC Biomarker on GSE14520 cohort for (A) OS, (B) RFS/DFS. [file Image_6.png]
